# Application of Culture-Independent Rapid Diagnostic Tests in the Management of Invasive Candidiasis and Cryptococcosis

**DOI:** 10.3390/jof1020217

**Published:** 2015-08-31

**Authors:** Michael A. Pfaller

**Affiliations:** 1 University of Iowa College of Medicine, Iowa City, IA 52242, USA; E-Mail: michael-pfaller@uiowa.edu; Tel.: +1-352-543-0362; 2 University of Iowa College of Public Health, Iowa City, IA 52242, USA; 3 T2 Biosystems, Lexington, MA 02421, USA

**Keywords:** candidasis, cryptococcosis, rapid diagnosis, invasive fungal infection

## Abstract

The diagnosis of invasive candidiasis (IC) and cryptococcosis is often complicated by slow and insensitive culture-based methods. Such delay results in poor outcomes due to the lack of timely therapeutic interventions. Advances in serological, biochemical, molecular and proteomic approaches have made a favorable impact on this process, improving the timeliness and accuracy of diagnosis with resultant improvements in outcome. This paper will serve as an overview of recent developments in the diagnostic approaches to infections due to these important yeast-fungi.

## 1. Introduction

The frequency of fungal disease, particularly that caused by systemic and opportunistic pathogens, has increased substantially during the past several decades [1,2,3,4,5,6,7,8,9,10,11,12]. This increase is primarily due to expanding patient populations at high risk for the development of opportunistic life-threatening fungal infections, which includes persons with AIDS, neoplastic disease, extremes of age, immunosuppressive therapy, and those undergoing organ transplantation (both hematologic and solid organ) and aggressive surgery [5,9,10,13,14,15,16,17,18,19,20,21,22,23,24,25,26,27,28,29,30]. Invasive fungal infections (IFI) in these populations are clearly important causes of morbidity and mortality. Serious infections are being reported with an ever-increasing array of pathogens (Table 1) not the least of which are the opportunistic yeast pathogens *Candida* and *Cryptococcus* species [9,24,31,32].

**Table 1:** Spectrum of opportunistic fungal pathogens ^a^.

Organism Group	Examples of Specific Pathogens	
***Candida***	*C. albicans*	*C. krusei*
	*C. glabrata*	*C. lusitaniae*
	*C. parapsilosis*	*C. guilliermondii*
	*C. tropicalis*	*C. rugosa*
***Cryptococcus***	*C. neoformans C. gattii*	-
**Other yeasts**	*Saccharomyces* species	*Rhodotorula* species
	*Trichosporon* species	*Malassezia* species
	*Blastoschizomyces capitatus*	-
***Aspergillus***	*A. fumigatus*	*A. versicolor*
	*A. flavus*	*A. terreus*
	*A. niger*	*A. calidoustus*
**Mucormycetes**	*Rhizopus* species	*Apophysomyces* species
	*Rhizomucor* species	*Cunninghamella bertholletiae*
	*Mucor* species	*Saksenaea* species
	*Lichtheimia (Absidia)* species	-
**Other hyaline**	*Fusarium* species	*Trichoderma* species
**Molds**	*Sarocladium (Acremonium)* species	*Purpureocilium (Paecilomyces) lilacinus*
	*Scedosporium* species	*Chrysosporium species*
**Dematiaceous**	*Alternaria* species	*Cladophialophora species*
**Molds**	*Bipolaris* species	*Phialophora* species
	*Exophiala* species	*Dactylaria* species
	*Ramichloridium* species	*Wangiella* species
**Dimorphic**	*Histoplasma capsulatum*	*Sporothrix schenckii*
**PLMolds**	*Coccidioides immitis/posadasii*	*Talaromyces (Penicillium) marneffei*
	*Blastomyces dermatitidis*	-
	*Paracoccidioides brasiliensis*	-
**Other**	*Pneumocystis jirovecii*	-
	Microsporidia species	

^a^ List not all inclusive.

Yeast-like fungal pathogens constitute the most common etiologic agents of IFI, the vast majority of which are due to *Candida* spp., *Cryptococcus neoformans* and *Cryptococcus gattii*. Invasive candidiasis (IC) is manifested by candidemia and deep-seated candidiasis (involving normally sterile body fluids and tissues) and cryptococcosis includes cryptococcal meningitis (CM), cryptococcemia, and cryptococcal pneumonia. Whereas most cases of IC are nosocomial or healthcare-associated (HCA) in origin, cryptococcal infections are generally considered to be community-acquired secondary to environmental exposure. Both IC and cryptococcosis occur predominantly in highly immunocompromised individuals (AIDS, solid organ transplant, neutropenia, malignancy, surgical trauma, steroids and other immunosuppressive medications); however, *C. gattii* frequently causes central nervous system (CNS) and pulmonary infections in apparently immunocompetent individuals and increasingly infection with *C. neoformans* is detected in individuals without demonstrable immunodeficiency [33]. Despite the availability of several antifungal agents with demonstrated activity against *Candida* spp., and *C. neoformans*/*gattii*, mortality due to these pathogens remains unacceptably high [1,5,34]. Aside from the negative impact of underlying disease conditions on mortality, delayed recognition of infection and subsequent administration of inappropriate therapy is increasingly recognized as a contributing factor to poor outcomes in both IC and cryptococcosis [1,33,34]. Given the lack of specific clinical signs and symptoms for these infections, diagnosis most often depends upon the results of established laboratory methods including microscopy, histopathology, serological and microbiological methods (Table 2). In many instances these methods are recognized to be insensitive, non-specific, or too slow to allow for prompt etiologic diagnosis. Fortunately, recent advances in immunological, biochemical, molecular and proteomic methods provide new approaches for both rapid and accurate species identification as well as direct detection of these fungal pathogens in clinical specimens (Table 2).

The conventional microbiological and histopathological methods for addressing the diagnosis and species identification of *Candida* and cryptococci are well covered in various reference manuals and documents [35,36,37,38] and will not be addressed in this review. This paper provides an overview of the newer culture-independent methods (*i.e.*, immunologic, biochemical, proteomic and molecular methods) for both identification and detection of these important fungal pathogens (Table 2).

**Table 2:** Laboratory diagnosis of invasive fungal infections.

Method	Specific Examples
A. Conventional Microbiologic	Direct microscopy (Gram, Giemsa, and Calcofluor stains) Culture Identification Susceptibility testing
B. Histopathologic	Conventional microscopy Routine stains (H&E) Special stains (GMS, Mucicarmine, PAS) Direct immunofluorescence In situ hybridization
C. Immunologic	Aspergillus lateral flow device (LFD) antigen test Blastomyces antigen test Cryptococcal antigen tests (LA,EIA,LFD) Histoplasma antigen test Galactomannan test Mannan/ antimannan test Antibody detection
D. Molecular and Proteomic Methods	Direct detection Identification Strain typing
E. Biochemical	Cell wall components (β-d-glucan)

^a^ Abbreviations: H&E, hematoxylin and eosin; GMS, Gomori’s methenamine silver; PAS, periodic acid-Schiff; LA, latex agglutination; EIA, enzyme immunoassay.

## 2. Laboratory Diagnosis

Prior to discussing the newer approaches to the detection and identification of *Candida* and *Cryptococcus* spp., it is useful to briefly consider some of the deficiencies of routine microbiological approaches to these infections, specifically regarding the culture of blood and cerebrospinal fluid (CSF).

## 3. Culture

The most sensitive means of diagnosing a fungal infection is generally considered to be the isolation of the infecting agent on culture media. Having said this, false-negative cultures are well documented in the face of disseminated fungal infection and even when positive, the results may be delayed or difficult to interpret [39,40,41,42,43,44,45]. In most instances culture is necessary to specifically identify the etiologic agent and, if indicated, to determine the in vitro susceptibility to various antifungal agents.

Although not all serious fungal infections are marked by hematogenous dissemination and fungemia, detection of fungemia is useful in diagnosing opportunistic infection due to *Candida* spp., *C. neoformans*, *C. gattii* and other yeasts such as *Trichosporon* spp. and *Malassezia* spp. [46,47,48]. Blood cultures (BC) may be negative in the face of disseminated disease [44,45]; however, advances in BC technology have markedly improved the ability of laboratories to detect fungemia [49,50,51]. The lysis-centrifugation method (Isolator, Wampole Laboratories, Cranbury, NJ, USA) and the continuous monitoring automated BC systems are all widely employed methods for the detection of fungemia due to *Candida* spp. [49,50]. The development of specialized broth media containing lytic agents, resins, charcoal, or diatomaceous earth coupled with continuous agitation has contributed to the improved performance of the broth-based systems [48,49,50,51,52,53,54,55]. However, recovery of *C. glabrata*, *C. neoformans* and *C. gattii* may be inferior with broth-based systems compared with the lysis centrifugation method [48,49]. On the other hand, culture contamination occurs more frequently with the more labor-intensive lysis centrifugation method.

Among the automated, continuous-monitoring BC systems that have been developed, the Bactec (Becton-Dickinson, East Rutherford, NJ, USA) and the BactT/Alert (bioMerieux, San Diego, CA, USA) systems are superior in their capability to recover yeasts from blood. Studies have demonstrated that these systems match the performance of the lysis centrifugation method for the detection of *Candida* spp. and *C. neoformans* [51,55]. It has been proposed that optimal detection of fungemia requires the collection of adequate volumes of blood (30–60 mL) and the use of both a broth- (vented, agitated) and an agar-based (lysis centrifugation) BC method [56]. It should be noted that the Bactec aerobic BC bottle and medium are notorious for poor recovery of *C. glabrata* and requires the use of a fungal medium (MycoF-lytic) for optimal recovery of this important species [48,52,53,57,58].

BCs remain an established approach to the diagnosis of IC [59,60,61,62]. This is despite the fact that BCs have always been considered to be too slow and insensitive to serve as an early diagnostic method [39,45,63,64]. It is increasingly apparent that as many as one-third of patients with IC never have a positive BC and even when positive, species-level results may not be available for 48 to 72 h or longer [45,65,66]. Notably, in a review of 415 autopsy-proven cases of IC, Clancy and Nguyen [45] demonstrated that BCs were positive in only 38% of cases. This low yield of BCs was confirmed by Avni *et al.* [44] who reported a pooled BC positivity rate of 38% in a meta-analysis of 10 studies for the diagnosis of IC. Cultures of blood and other clinical specimens may be rendered falsely negative by the use of prophylactic or empiric antifungal therapy further confounding efforts to establish a firm diagnosis [58]. Despite these negative features, BCs remain at the heart of care guidelines for IC in hospitalized individuals [59,61]. It is now understood that the information required to limit mortality and the emergence of antifungal resistance is time-critical and as such rapid culture-independent diagnostic tests are required to complement BCs in the management of high-risk patients [39,45,63].

BCs are positive in 30%–50% of patients with cryptococcal meningitis (CM) [33]. Among patients with suspected CM, CSF cultures are positive in 90% of cases and as with BCs the yield improves when larger volumes of CSF are cultured (Table 3) [67]. Quantitative cultures of CSF provide information of both prognostic and therapeutic value. The viable quantitative CSF yeast count provides an estimate of the fungal burden and serial quantitative yeast measurements during antifungal therapy can be used for therapeutic monitoring by determining the early fungicidal activity of the treatment regimen [33,68].

## 4. New Approaches to the Identification of *Candida* and *Cryptococcus* Species

Identification of fungi to genus and species is increasingly important as the spectrum of opportunistic pathogens continues to expand [3,6,7] (Table 1). Although the clinical presentation of many fungal infections may be indistinguishable, specific identification of the etiologic agent may have a direct bearing on the management of the infectious process. It is increasingly apparent that one cannot rely on a single therapeutic approach (e.g., administration of amphotericin B) for the management of all, or even most, fungal infections [69,70,71,72]. Furthermore, the identification of fungal pathogens may have additional diagnostic and epidemiologic implications. In the case of the more unusual mycoses, specific etiologic identification may provide access to the literature and the experience of others regarding the probable course of infection and response to therapy.

**Table 3:** Performance characteristics of cryptococcal diagnostic assays on cerebrospinal fluid (CSF) in persons with suspected meningitis ^a^.

Diagnostic test ^b^	No. samples	Sensitivity (%)	Specificity (%)	PPV (%)	NPV (%)
CRAG LFA	666	99.3	99.1	99.5	98.7
CRAG latex (Meridian)	279	97.8	85.9	92.6	95.5
CRAG latex (Immy)	749	97.0	100.0	100.0	75.8
India Ink microscopy	805	86.1	97.3	98.2	80.2
CSF culture	806	90.0	100.0	100.0	85.3
—100 µL volume	524	94.2	100.0	100.0	91.2
—10 µL volume	282	82.4	100.0	100.0	75.8

^a^ Data compiled from Boulware *et al.* [67]. ^b^ Abbreviations: CRAG, cryptococcal antigen; LFA, lateral flow assay; CSF, cerebrospinal fluid; PPV, positive predictive value; NPV, negative predictive value.

## 5. Phenotypic Methods

Since *C. albicans* constitutes the vast majority of yeasts recovered from clinical specimens, several rapid and simple tests have been devised to distinguish it from other yeasts [36,73,74,75,76,77,78,79,80,81,82]. The most widely used test for identification of *C. albicans* is the germ tube test [38,83]. *C. albicans* forms germ tubes within 3 h when incubated in serum or plasma at 35 °C. Other species of *Candida* are capable of germ tube formation but require extended incubation. *C. dubliniensis* and *C. stellatoidea* are capable of forming germ tubes within 3 h and may be difficult or impossible to differentiate from *C. albicans* without performing additional physiologic, immunologic, or nucleic acid-based testing [36,80,81,84,85].

Chromogenic media, such as CHROMagar, and rapid (<24 h) colorimetric tests based on the detection of *C. albicans-*specific enzymes (l-proline aminopeptidase and β-galactose-aminidase), have proven useful in the rapid presumptive identification of *C. albicans* [38]. Although a single presumptive identification test is not sufficient for identifying most yeasts, a positive germ tube or colorimetric test or characteristic green colony on CHROMagar medium is generally considered to be acceptable for the identification of *C. albicans* [38,76].

Among the more than 200 species of *Candida* that have been identified, five—*C. albicans*, *C. glabrata*, *C. parapsilosis*, *C. tropicalis*, and *C. krusei*—account for 95%–98% of cases of IC [4,8,9]. Recent reports indicate that shifts have occurred in the distribution of non-*albicans* species with the emergence of *C. glabrata*, *C. krusei*, *C. lusitaniae*, and other less common species [3,6,9,32,86,87]. Infections with these various species may require different therapeutic considerations [46,48,61,69,70,71,72,88,89] and so further identification of all germ tube-negative or colorimetric test-negative yeasts is mandatory for isolates obtained from blood and other normally sterile body fluids [61,88,90,91]. Due to the pathogenic potential of *C. neoformans/gattii*, all encapsulated yeasts from any body site should also be identified. There are several rapid screening tests that may be used for the presumptive identification of *C. neoformans* including the urease test (positive), nitrate test (negative) and production of phenol oxidase (positive) [38]. There is a simple biochemical determination that allows the differentiation of *C. neoformans* from *C. gattii*. A canavanine-bromthymol-glycine agar plate assay allows the separation of the two species by the color of the colonies [38]. More recently application of molecular and proteomic methods have greatly expanded the abilities of the clinical laboratory to identify *Candida* and cryptococci to species level [36,39].

Further identification of *Candida* and cryptococci to species requires the determination of biochemical and physiologic profiles as well as an assessment of their morphology when grown on a medium such as cornmeal agar or yeast morphology agar [36,38,83]. In addition to the identification of *C. albicans*, colony morphology on CHROMagar allows the presumptive identification of *C. tropicalis* and *C. krusei* [73,74]. Likewise, *C. glabrata* may be identified by a rapid trehalose test [38] or differential growth on blood agar (no growth or slow growth) versus eosin methylene blue agar (rapid growth) [92]. Carbohydrate assimilation tests provide definitive identification for most *Candida* and *Cryptococcus* species and may be performed by using one of several commercial identification systems [36,38]. Differentiation of yeasts with similar biochemical profiles can usually be accomplished by observing their microscopic characteristics on cornmeal agar [38,83].

## 6. Proteomic Methods

One of the most important advances in the post-culture identification of fungi is that of matrix-assisted laser desorption ionization-time of flight (MALDI-TOF) mass spectrometry (MS) (Table 4) [93,94]. MALDI-TOF MS uses species-specific patterns of peptides and protein masses to identify microorganisms. It has been shown to be highly accurate in identifying a broad array of bacteria and recently has been shown to provide a rapid and reliable method for the identification of yeasts, yeast-like fungi and some molds [94]. The technique involves the use of whole cell preparations or an extract of proteins from the fungal cells, spotting of the specimen on a grid and overlaying the spot with a matrix. The proteins are ionized by a laser and migrate through a charged field in a vacuum tube towards a detector. The spectrum is generated rapidly (~10 min per specimen) and is compared to a reference database. Presently there are two commercial systems based on this method available in the U.S. that are able to identify yeast and mold species. Studies evaluating their performances are promising, showing that this method is able to accurately and rapidly identify *Candida* spp. and *Cryptococcus* spp., including *C. gattii*, from positive cultures, with a high concordance (>90%) in comparison to both conventional and molecular methods [39,94]. In some instances, MALDI-TOF MS has been shown to be superior to conventional methods [94].

**Table 4:** Commercial molecular assays for detection and identification of *Candida* and *Cryptococcus* ^a^.

Assay	Manufacturer	Method	Detectable Pathogens	Detection limit (CFU/ mL)	Turnaround time (h)
*Performed On Positive Blood Culture Bottles*					
Luminex xTAG Fungal ASR Assay	Luminex Corp. Austin, TX, USA	Multiplex PCR and bead-based flow cytometry	Identification of 23 different fungi including *Candida* and *Cryptococcus*	NA	5–6
PNA-FISH	AdvanDX, Woburn, MA, USA	Fluorescence-based hybridization with PNA probes	Identification of 5 *Candida* species	NA	<1
FilmArray	Idaho Technology, Salt Lake City, UT, USA	Multiplex PCR	Identification of 5 *Candida* spp.	NA	1
Prove-it Sepsis	Mobidiag, Helinski, Finland	Multiplex PCR with hybridization on a microarray	Identification of 13 fungi including *Candida* and *Cryptococcus*	NA	3–5
MALDI-TOF MS	Brucker Daltonics, Bremer, Germany bioMerieux, Marcy l’Etoile, France	Mass spectroscopy	Identification of many fungi including *Candida* and *Cryptococcus*	NA	<1
*Performed Directly On Whole Blood*					
SepsiTest	Molzym, Breman, Germany	Broad-range PCR with sequencing	Identification and detection of 5 species of *Candida* and *Cryptococcus*	20–40	8–12
Vyoo	SIRS- Lab, Jena, Germany	Multiplex PCR with gel electrophoresis	Identification and detection of 6 species of *Candida*	3–10	6–8
Plex-ID	Abbott, Carlsbad, CA, USA	Multiplex PCR detected by electrospray ionization mass spectroscopy	Identification and detection of many fungi including *Candida* and *Cryptococcus*	3–16	6–8
Magicplex Sepsis Real-Time Test	Seegene Inc., Seoul, South Korea	Multiplex real-time PCR	Identification and detection of 6 species of *Candida*	NA	3–4
Real-Time PCR Panel	Quest Diagnostics, Madison, NJ, USA	Multiplex real-time PCR	Identification and detection of 5 species of *Candida*	1–350	6
Real-Time PCR Panel	Viracor-IBT Laboratories, Lee’s Summit, MO, USA	Real-time PCR	Identification and detection of 5 species of *Candida*	<1	6
LightCycler SeptiFast Test	Roche Molecular Systems, Branchburg, NJ, USA	Multiplex real-time PCR	Identification and detection of 5 species of *Candida*	30–100	6
T2Candida Panel	T2 Biosystems, Lexington, MA, USA	PCR with nanoparticle capture and T2 magnetic resonance detection	Identification and detection of 5 species of *Candida*	1–3	3–5

^a^ Abbreviations: NA, not available; PCR, polymerase chain reaction; PNA-FISH, peptide nucleic acid-fluorescent in situ hybridization; MALDI-TOF MS, matrix-assisted laser desorption ionization-time-of-flight mass spectrometry.

## 7. Molecular Methods

The use of both nucleic acid probes and amplification-based molecular approaches provide more rapid and objective identification of fungi compared with traditional phenotypic methods [39,95,96,97,98,99].

Among the newer rapid, post-culture methods for identification of *Candida* are the techniques of peptide nucleic acid (PNA)—fluorescence in situ hybridization (FISH) [39]. The PNA FISH tests (AdvanDx, Woburn, MA) are based on a fluorescein-labeled PNA probe that specifically detects *C. albicans*, *C. tropicalis*, or *C. glabrata* as individual species or detects a yeast species group (e.g., *C. albicans* and *C. parapsilosis* fluoresce green, *C. tropicalis* fluoresces yellow and *C. glabrata* and *C. krusei* fluoresce red with the Yeast Traffic Light™ PNA FISH kit (AdvanDx, Woburn, MA, USA)) in blood cultures by targeting species-specific rRNA sequences. The probes are added to smears made directly from the contents of the blood culture bottle and are hybridized for 90 min. Recent modifications to the probes and reagents have resulted in a second generation test (*Quick*FISH™, AdvanDx, Woburn, MA, USA) that shortens the assay time to 30 min. Smears are subsequently examined by fluorescence microscopy. The test has been shown to have excellent sensitivity (99%), specificity (100%), positive predictive value (100%) and negative predictive value (99.3%) [100].The use of PNA-FISH may provide a time savings of 24 to 48 h, compared with conventional laboratory methods used for identification. It allows physicians to be notified of the yeasts identity along with positive blood culture results. Rapid, accurate identification of *C. albicans*, *C. tropicalis*, *C. parapsilosis*, *C. glabrata* and *C. krusei* should promote optimal antifungal therapy with the most cost-effective antifungal agents, resulting in improved outcomes and significant antifungal savings for hospitals.

Amplification-based methods are increasingly being applied to the post-culture identification of yeasts and molds (Table 4) [93,94,95,97,98,99,101,102]. Both ribosomal targets and internal transcribed spacer (ITS) regions have proven useful for the molecular identification of a wide variety of fungi. A major limitation of this approach is the variable quality and accuracy of the existing sequence databases [99]. Presently, with the availability of improved sequencing techniques, broader and more reliable databases, and more readily available kits and software, this technology has become a competitive alternative to the classic mycological identification methods used for clinically important fungi [34,36,96,97,103,104].

Multiplex PCR platforms have been developed for the identification of *Candida* and *Cryptococcus* species in postitive BC samples [101,105,106]. The FilmArray™ BC identification panel (BioFire, Salt Lake City, UT, USA) and the xTAG™ fungal analyte-specific reagent (ASR) assay (Luminex Molecular Diagnostics, Toronto, Canada) have demonstrated sensitivities and specificities of 100% and 99%, respectively, for the five most common species of *Candida* [39,101]. The FilmArray™ approach provides fully automated nucleic acid extraction, amplification and detection and has been FDA cleared for post-culture identification of bacteria and *Candida* species. Likewise the xTAG ASR approach has been shown to provide not only accurate species-level identification of *C. neoformans* and *C. gattii* but also has been used to determine specific genotypes within these species which has both epidemiological and prognostic utility [105,106]. In addition to these methods available in the U.S., there are additional molecular platforms for post-culture identification of yeast species available in Europe (Table 4). Thus molecular methods for fungal identification from positive cultures improve the time to identification when compared to conventional methods of identification and may be useful in antimicrobial stewardship interventions [107,108]. The disadvantages of these post-culture approaches is that they still depend on a positive culture, which may be negative in >50% of cases of IC and cryptococcosis [44,45]. Pan-fungal nucleic acid amplification test (NAAT) platforms are emerging and offer promise for an expanded menu of fungal targets [39].

## 8. Immunologic, Biochemical and Nucleic Acid-Based Methods of Diagnosis

Although culture and histopathology remain the primary means of diagnosing fungal infections, there continues to be a need for more rapid, nonculture methods for diagnosis [34,36,39,40,41,42,109,110,111,112,113,114]. Tests for detection of antibodies, rapid detection of specific fungal antigens and cell wall components, and fungal species-specific RNA or DNA sequences have the potential to yield rapid diagnostic information that can guide the early and appropriate use of antifungal therapy [34,39,43,64,109,112,113]. Although a great deal of progress has been made in these areas, the true impact on the diagnosis and outcome of invasive fungal infections (IFI) has yet to be realized [39,63,101,110].

## 9. Antibody Detection

Serologic tests can provide a rapid means of diagnosing fungal infections, as well as a means to monitor the progression of the infection and the patient’s response to therapy by comparing serial determinations of antibody or antigen titers [34,109,115,116,117,118]. Most conventional serologic tests are based on detection of antibodies against specific fungal antigens. Often, this serodiagnostic approach is ineffective, because many patients who are at risk for IFI are not capable of mounting a specific antibody response to infection. In addition, determination of the presence of an acute infection typically requires a comparison of the type and quantity of antibody present in both acute-phase and convalescent-phase serum samples, an exercise that is not helpful during the acute presentation, when therapeutic interventions are being decided [34,109].

Antibody tests for *Candida* and *Cryptococcus* spp. may be performed; however, these tests are frequently unable to distinguish between active and past infection on the one hand and colonization on the other [115,116]. Furthermore, a negative serologic test does not rule out infection because immunocompromised patients and some individuals with disseminated infection may not mount an antibody response to the infecting organism.

Several commercial enzyme-linked immunosorbent assay (ELISA) techniques are now available which detect anti-*Candida* antibodies in an effort to improve the diagnosis of IC [115,116,119,120]. These kits have shown sensitivities ranging from 50% to 90% and specificities of ~15% to 65% [115]. The evaluation of an ELISA-based kit (Syscan 3; Rockeby, Biomed Ltd, Singapore, Singapore) for detection of anti-*Candida* antibodies (anti-enolase and intracytoplasmic antigens) demonstrated a sensitivity, specificity, positive predictive value, and negative predictive value for IC of 74%, 75%, 62%, and 84% in a group of immunocompetent patients and 15%, 60%, 1.7%, and 93% in an immunocompromised group [116]. Despite a moderately high negative predictive value it is difficult to see how such testing would be of much value especially among high risk patients.

Other groups have reconsidered the value of detection of anti-mannan antibodies in the diagnosis of IC. The Platelia *Candida* antibody test (Bio-Rad, Redmond, WA, USA) uses an ELISA format to capture circulating anti-mannan antibodies in sera from patients, with reported specificity and sensitivity values of 94% and 53%, respectively [119]. When performed simultaneously in combination with a mannan antigen detection test, the method gave a sensitivity of 80% and a specificity of 93% [119,120]. Other authors showed a sensitivity and specificity of 59% and 63%, respectively for the Platelia anti-mannan test with an improved sensitivity of 95% and a lower specificity of 53%, when combined with a test for mannanemia [115]. It appears from these results that the diagnosis of IC cannot be made using a single test for antibodies alone. Rather, a strategy based on detection of mannanemia and anti-mannan antibodies may prove to be the most useful [115,118]. In a recent meta-analysis, Mikulska *et al.* [121] reported a combined mannan/anti-mannan sensitivity and specificity for candidiasis diagnosis of 83.0% and 86.0%, respectively: separate sensitivities and specificities of 58% and 93%, respectively, for mannan antigen alone and 59% and 83%, respectively, for anti-mannan antibodies alone. Furthermore, it appears that regular (at least twice weekly) serum sampling is critical to achieving an early diagnosis of IC [115,119,120,121,122]. It should be noted that the findings of Mikulska *et al.* [121] may be limited by the heterogeneity in the studies evaluated in the meta-analysis.

Antibodies (IgG, IgM, IgA) to the polysaccharide capsule of *C. neoformans* can be detected in patients with cryptococcosis [33,38]. The utility in diagnosis is limited although antibody response may have prognostic significance and can be a marker of reactivation of infection in solid organ transplant recipients [33].

## 10. Immunologic and Biochemical Antigen Detection Methods

Tests to detect fungal antigens in serum or other body fluids represent a direct means of providing a serodiagnosis of IFI [34,39,114,118,119,120,123,124,125,126,127]. Significant advances have been made in recent years [1,2,4,60,75]; however, for most fungal infections a widely acceptable method is not available. Although several tests for the detection of fungal antigens have been standardized and are now available commercially, issues still remain concerning the sensitivity and specificity of the various tests in certain patient populations, which populations should be monitored, how often should testing be performed, how the test behaves over time in relation to disease progression or improvement, what testing strategies are the most practical and cost-effective, and what is the true impact of such testing on patient outcome [128,129]?

Presently, the most established and widely used fungal antigen tests are the latex agglutination (LA) and enzyme immunoassay (EIA) tests for the detection of the capsular polysaccharide antigen of *C. neoformans/gattii* in CSF and serum. The commercially available tests for cryptococcal antigen detect >95% of cryptococcal meningitis and approximately 67% of disseminated cryptococcal infections [123]. These antigen tests are well standardized, widely available and supplant India Ink for the diagnosis of cryptococcal meningitis [123]. A newly developed method to detect cryptococcal antigen employs a lateral-flow device (LFD) immunoassay and has been shown to provide results that are comparable to those of EIA and LA methods in both serum and CSF (Table 3) [39,130]. The low cost, ease of use, and high degree of accuracy of the LFD make it very promising as a point-of-care diagnostic method for use in both low- and high-resource settings [131,132].

Mannan is the major circulating antigen in patients with IC. Detection of mannan is complicated by rapid clearance from the patient’s sera and binding by anti-mannan antibody. Although circulating mannan may be detected by several methods, a dissociation of antigen-antibody complexes is required for optimal sensitivity [43]. Sensitivities of 25%–100% and specificities of 92%–100% have been reported with EIA assays for mannan detection [43,112,118,119,120,124,127,133,134,135]. An early commercial system to detect mannan used a LA format (Pastorex *Candida* test, Sanofi Diagnostics Pasteur, Mames-la-Coquette, France) and demonstrated poor sensitivity (0%–25%) due to the rapid clearance of mannan from patients’ sera and the insensitive LA format [136]. More recently, the Platelia *Candida* antigen test (BioRad) uses a monoclonal antibody-based double sandwich EIA format with a resultant increase in sensitivity and a limit of detection of 0.1 ng of mannan per mL of serum [118,119,120]. Clinical evaluations of the Platelia *Candida* antigen test report sensitivities ranging from 40% to 86% and specificities from 79% to 98% [112,119,120,121,124,133]. Virtually every study evaluating the detection of the mannanemia has shown that multiple serial samples are required to overcome the rapid clearance of mannan from patient’s sera and to optimize diagnostic sensitivity.

Sendid and colleages [118,119,120] have investigated the use of the Platelia *Candida* anti-mannan antibody test in combination with the Platelia *Candida* antigen test to maximize the ability to diagnose IC. As discussed previously, simultaneous testing for mannanemia and anti-mannan antibodies resulted in an improved sensitivity from 40% to 53% with the single tests to 80% with combined testing [119,120,121]. Specificity remained high (93%) with the combined testing strategy. Whereas the Platelia *Candida* antigen test is specific for α-mannan, the Platelia antibody test detects antibodies against the whole mannan oligomannose repertoire containing both α- and β-mannan epitopes [119,127]. These and other investigators emphasize the importance of regular (twice weekly) serial monitoring of at-risk patients in maximizing the sensitivity of serodiagnostic testing for IC [115,119].

A simple and commercially available test (CAND-TEC *Candida* Detection System, Ramco Laboratories, Houston, TX, USA) relies on the detection of a structurally uncharacterized 56 °C heat-labile antigen of *C. albicans* using an LA format [137]. Although easy to perform, the test suffers from low sensitivity (as low as 0%–16%) and specificity, and its usefulness for the reliable diagnosis of IC is limited [109,124,134,138].

Several different protein antigens of *Candida* have been explored for their diagnostic potential [43,109]. These antigens include secreted aspartyl proteinases (SAPs), *C. albicans* heat shock protein (hsp) 90, and enolase [134,139,140,141]. One confounding factor that all of these protein antigens share is the fact that they form antigen-antibody immune complexes, which negatively impacts the sensitivity of antigen detection assays.

Detection of other compounds released by fungal cells during infections has also been explored for the diagnosis of IC [39,109,142]. The most promising among these various targets is detection of serum β-d-glucan (BDG; for a non-specific fungal diagnosis including IC). BDG is an important component of the cell wall of *Candida*, *Aspergillus*, *Pneumocystis* and many other pathogenic fungi. Although BDG is not immunogenic, the fact that it can be found circulating in the bloodstream of patients with IFI has been exploited for use diagnostically and as a surrogate marker of infection [125,126,133,134,143,144]. The Fungitell BDG assay (Associates of Cape Cod, Inc., Falmouth, MA, USA), is an FDA cleared commercially available colorimetric assay that can indirectly determine the concentration of BDG in the serum. The detection system is based on the activation of a BDG-sensitive proteolytic coagulation cascade, the components of which are purified from the horseshoe crab [145]. The assay can measure picogram amounts of BDG and has been used to demonstrate the presence of the polysaccharide in the serum of patients with IC and IA, but not cryptococcosis or mucormycosis (organisms lack BDG) [125,133,134,142,143,144]. Several studies have demonstrated a modest degree of sensitivity and specificity in the diagnosis of IC (78%–97% sensitivity and 88%–100% specificity) [125,126,133,134,142,146].

The Fungitell BDG assay has been evaluated for the early diagnosis of IFI in patients with hematologic malignancies [125] and in a multicenter study of patients with IFIs and healthy control subjects [146]. In the latter study sera was obtained from 170 fungal infection-negative control subjets and from 163 patients with proven or probable IFI diagnosed at one of 6 participating medical centers. Overall the sensitivity and specificity of the assay were 69.9% and 87.1%, respectively, with a PPV of 83.8% and a NPV of 75.1%. The sensitivity of the BDG test was 81.3% among the 107 patients with proven IC. Measurement of serum BDG was considered to be a useful diagnostic adjunct for IFI. Another study of patients with acute myelogenous leukemia suggested that the sensitivity, specificity, and PPV of the assay increased significantly if sera were obtained twice weekly [125]. Obtaining multiple samples increased the sensitivity, PPV, and NPV of the BDG assay to >98% for subjects with leukemia who were receiving antifungal prophylaxis. A recent meta-analysis of 16 studies measuring serum or plasma BDG for the diagnosis of IC reported pooled sensitivity and specificity values of 75.3% and 85.0%, respectively [142]. Although the BDG test may be performed in a hospital-based laboratory, it is a send-out test for most institutions resulting in a turn around time for results measured in days rather than hours. Regarding on-site BDG testing, the assay has been adapted to an automated coagulation format that could potentially be used to generate results in the clinical laboratory and avoid the delays of send-out testing. Such an approach would require extensive standardization and validation before being used clinically.

In contrast to these rather favorable studies, other investigators have pointed out considerable problems with the specificity of the BDG assay for IFI [126,147]. Pickering *et al.* [126] tested sera from healthy blood donors and patients with candidemia and found a sensitivity and specificity of 92.9% and 100%, respectively. When bacteremic patients were included in their assessment of the performance of the BDG assay, the specificity and PPV fell to 77.2% and 51.9%, respectively, due to a high number of false-positive results, especially in samples from patients with Gram-positive bacteremia. These investigators demonstrated that excess manipulation of a sample can result in contamination by BDG [126]. They also found that hemolysis would cause false-positive BDG test results and that high concentrations of bilirubin and triglycerides were inhibitory and would cause false-negative results. These confounding factors can be added to a list of other causes of false-positive BDG test results including hemodialysis with cellulose membranes, patients treated with intravenous immunoglobulins, albumin, coagulation factors, or plasma protein factor, or patients exposed to gauze or other materials that contain glucans [126]. Thus a negative BDG test result may be useful for ruling out an IFI due to most fungal pathogens with the exception of cryptococci or the Mucormycetes; however, a single positive result should be confirmed by testing another specimen (two consecutive positive tests) and a thorough review for potential sources of false positivity should be conducted.

## 11. Nucleic Acid Detection

As in other areas of microbiology, the application of molecular biology, specifically the polymerase chain reaction (PCR), offers great promise for the rapid diagnosis of fungal infections [34,36,39,42,43,44,63,64,95,148,149,150,151]. At present, most of the research has been focused on the diagnosis of IC [42,43,44,109,112,148,149,150,152] and invasive aspergillosis (IA) [39,113,153,154,155,156,157,158,159,160]; however PCR has also been applied to the diagnosis of other IFI, including CM [36,39,42,95,105,106,156,161,162,163,164,165,166]. It should be noted, however, that despite a great deal of interest in molecular approaches to the diagnosis of infectious diseases, molecular methods are used in only 5% of laboratories providing diagnostic services in medical mycology [167]. Given the limitations of current fungal diagnostics, the use of PCR as a diagnostic adjunct for the diagnosis of IFI is especially promising; however, considerable additional research, standardization of testing protocols, and prospective assessment is necessary before one can conclude that molecular biology has achieved a tangible clinical pay-off [34,39,63,101,110].

PCR-based methods for the diagnosis of IFI have been applied to a variety of specimen types including whole blood, serum, tissue, BAL fluid, and CSF [39,42,43,44,63,95]. In addition to the use of whole blood or serum as the optimal specimen type, important procedural considerations need to be taken for removal of contaminating non-fungal DNA, breaking fungal cells for DNA extraction, and prevention of introduction of contaminating fungal DNA as well as minimizing the destruction of target DNA [39,43,44,109,148]. Target sequences vary widely but include genus-and species-specific variable regions as well as highly conserved regions of the fungal genome [39,42,43,44,63,95]. Both single (e.g., *hsp*90, lanosterol demethylase, chitin synthase, actin, urease) and multicopy (e.g., ribosomal, intergenic transcribed spacer regions (ITS), mitochondrial) gene targets have been studied, although molecular diagnostic methods targeting multicopy genes generally have better sensitivity than those targeting single copy genes [39,43,44,148]. The use of multicopy ribosomal (18S rRNA, 28S rRNA, 5.8S rRNA) and ITS targets offer the potential for sensitive panfungal markers for detection of IFI, followed by identification at the genus or species level [39,42,43,44,95,148].

PCR amplicon detection methods vary widely but most laboratory developed tests (LDT; “home brew”) employ capture probes in an ELISA format. Recent developments such as real-time PCR, gene chip technology, and the coupling of nanotechnology with T2 magnetic resonance (T2MR) detection will facilitate the broad use of this technology [39,64,65,101,148,150].

Irrespective of the technology used, most reports in the literature indicate that the sensitivity of PCR-based diagnosis is equal to or better than other currently used diagnostic techniques [39,42,43,44,63,109,148]. The major impediment to the application of PCR to the diagnosis of IFI has been the lack of standardization and the need for nucleic acid extraction and purification from clinical samples [63,64]. The studies reported in the literature must be viewed with the caveat that the vast majority are derived from in-house LDT protocols developed by different groups of investigators using different samples (e.g., whole-blood versus serum), different protocols for sample preparation, different molecular targets, and different PCR detection platforms [39,42,43,44,63,109]. Thus when considering the use of PCR for the diagnosis of either IC or cryptococcosis, one encounters a range of sensitivities and specificities of 77% to 100% and 66% to 100% , respectively, for IC and of 92% to 100% and 100%, respectively, for cryptococcosis [39,44,160,166]. Among several reasons for the reported low levels of sensitivity include requirements for nucleic acid extraction and the use of optical methods of detection resulting in excessively high limits of detection (LOD) [39,63,101,148].

The lack of standardization makes any comparison among studies very difficult and further hinders efforts to perform prospective, multicenter clinical trials [44,63,109]. Thus, despite the availability of promising new approaches for the rapid diagnosis of IFI, we have little data from prospective, multicenter clinical trials of sufficient size to determine the performance characteristics of the diagnostic methods or the most cost-effective manner in which to use them [39,63].

PCR-amplified *Candida*-specific DNA has been recovered from blood and other body fluids obtained from infected patients (Table 4) [39,42,43,44,65,101,148,150,168,169,170,171,172]. The most frequently employed targets for the diagnosis of IC are the multicopy broad-range panfungal genes such as the 18S, 5.8S, and 28S ribosomal RNA genes (rRNA), and the intergenic transcribed spacer (ITS) regions within the rRNA gene cluster [39,42,43,44,65,95,148]. In a systematic meta-analysis evaluating PCR assays for the diagnosis of IC, pooled analysis of 54 studies and almost 5000 patients found that the optimal conditions for the detection of *Candida* in blood using PCR were (i) the use of whole blood, (ii) a multi-copy target (rRNA or P450 genes), and (iii) a limit of detection (LOD) of ≤10 CFU per mL of blood [44]. Under these conditions the pooled sensitivity and specificity of PCR in diagnosing IC was 95% and 92%, respectively [44]. Importantly, PCR-based tests for *Candida* DNA in blood are negative in most subjects with gastrointestinal colonization with *Candida* species and the specificity of these tests is quite high [44,112,149,150,151,173,174].

Aside from using a consensus process to develop a standardized LDT for the diagnosis of IC, standardization may also be achieved by either a centralized model whereby a molecular test is offered and performed by a publically available reference laboratory or by commercialization of a molecular test to be performed on-site [63,64,65,148,150]. Commercialization of molecular tests not only standardizes the method but also facilitates large-scale “real world” clinical validation, leading to the implementation of the molecular test for clinical use [63].

Presently there are several commercially available amplification-based molecular tests that are available for use in the diagnosis of IC directly from the clinical specimen (Table 4). However, there are only three that have been evaluated in any detail in either the United States (US; Viracor *Candida* Real-Time PCR Panel (Viracor-IBT) and the T2Candida Panel (T2Biosystems)) or Europe (LightCycler SeptiFast Test (Roche Diagnostics) and theT2Candida Panel (T2Biosystems)) [64,65,148,149,150]. Other direct from blood sample platforms that include *Candida* are commercially available outside the U.S. (Table 4): SepsiTest (Molzym, Bremen, Germany), MagicPlex (Seegene, Seoul, Korea), VYOO (SIRS-Lab, Jena, Germany), PLEX-ID (Abbott Molecular, Carlsbad, CA, USA). The published clinical evaluations of these methods are weighted heavily towards the detection and identification of bacteria and they are not included in this review [175,176,177,178,179,180,181,182].

The Viracor (Kansas City, MO, USA) reference laboratory offers a real-time PCR panel for the detection of the five major species of *Candida* (*C. albicans*, *C. tropicalis*, *C. parapsilosis*, *C. glabrata*, and *C. krusei*) in serum or plasma. This is an LDT test and has not been cleared by the FDA. The Viracor *Candida* Real-Time PCR Panel requires nucleic acid extraction and purification and reports an LOD of ≤1 CFU/mL in serum or plasma [65]. In a single center clinical evaluation, the sensitivity and specificity of the Viracor Panel for the diagnosis of IC was 80% and 70%, respectively [65]. Although the in-laboratory turn around time (TAT) for the Viracor Panel is reported to be within 6 to 8h, the send out nature of the test means that the actual TAT for reporting results to the clinician is measured in days rather than hours.

Another real-time PCR test for IC is offered in the US by Quest Diagnostics reference laboratories (Table 4) (Madison, NJ, USA). Similar to the Viracor *Candida* Real-Time PCR Panel, the Quest *Candida* DNA, Qualitative, Real-Time PCR test is not FDA-cleared, requires nucleic acid extraction and purification from serum and detects the five most common species of *Candida*. The LOD for the Quest *Candida* test is reported to be *≤*1 to 350 CFU/mL depending on the species. Thusfar the clinical sensitivity and specificity of this test has not been reported in the literature.

The LightCycler SeptiFast Test uses a real-time PCR platform and has been extensively evaluated for use in Europe but has not been cleared by the FDA for use in the US [64,149]. SeptiFast detects nucleic acids extracted from whole blood for five species of *Candida* as well as 19 species of bacteria and *Aspergillus fumigatus*. The LOD for *Candida* using SeptiFast ranges from 30 CFU/mL (*C. albicans*, *C. parapsilosis*, *C. tropicalis* and *C. krusei*) to 100 CFU/mL (*C. glabrata*) [149]. When performed in the laboratory the TAT for a single test is approximately 6h. In a meta-analysis Chang *et al*. [149] reported a pooled sensitivity of 61% and a specificity of 99% for the detection of *Candida* in whole blood following extraction.

The T2Candida Panel is an FDA-cleared rapid diagnostic approach that employs a proprietary formulation enabling inhibitor-free target amplification of the multicopy ITS2 (internal transcribed spacer region 2) region of the *Candida* genome coupled with detection nanoparticles coated with oligonucleotide capture probes to enable sensitive and specific detection of the amplified product directly in whole blood without the need for culture or nucleic acid extraction steps [148]. The T2Candida Panel uses T2MR relaxometry to measure the magnetic properties of the water molecules in the specimen (providing signal amplification and non-optical detection) and not just the amplified target to achieve high level of sensitivity in complex clinical samples [148]. Results from a formal limit of detection (LOD) study of the T2Candida Panel showed an LOD of 1 to 3 CFU/mL of blood (1 CFU/mL for *C. tropicalis* and *C. krusei*, 2 CFU/mL for *C. albicans* and *C. glabrata* and 3 CFU/mL for *C. parapsilosis*) with results available as fast as 3h compared to >48 h for BC (Table 4) [57,148]. In a recently completed 1801 subject clinical trial the T2Candida Panel showed a sensitivity and specificity for the detection of candidemia of 91.1% and 99.4%, respectively [150]. Importantly the median time for species identification in this trial was 4.4 h for T2Candida and 129 h for BC. Likewise the median time for a negative result was 4.2 h for T2Candida and >120 h for BC. The T2Candida Panel allows for the identification of the five major species of *Candida* (*C. albicans*, *C. tropicalis*, *C. parapsilosis*, *C. glabrata* and *C. krusei*). The test is completely automated and requires <5 min of hands-on time.

PCR methods have also been developed and evaluated for the detection of *C. neoformans* and *C. gattii* from CSF and serum [105,106,160,166]. Whereas these methods are reported to be both sensitive (range 92.9% to 100%) and specific (100%), clinical implementation is unlikely given the highly sensitive, specific and simple methods currently available for detection of cryptococcal antigen in CSF and serum (Table 3) [33,67].

The changing epidemiology of IFI and the increasing recognition of the pathogenic potential of a bewildering array of fungal species in the high-risk patient population (Table 1), underscores the need for diagnostic testing approaches to detect a wide range of medically important fungi [5,7,34,39,109]. Thus, the application of broad-range (panfungal) PCR to amplify a target (e.g., 18S rRNA) from all or most common opportunistic fungi is an important strategy [95,110,156,157,164,165,183]. This approach combined with hybridization using specific or panfungal oligonucleotide probes enables the detection of pathogenic fungi with an analytical sensitivity of 1–10 fg (femptogram) of DNA per mL [95]. Universal primer pairs can specifically amplify the 18S rRNA gene segment from several medically important fungal pathogens including *Candida* species, *Aspergillus* species, *Trichosporon* species, *Fusarium* species, and *Cryptococcus* species, but not from bacteria or human samples. Real-time PCR techniques combined with automated DNA extraction methods [184] and panfungal primers enable the detection of several different pathogens in a single reaction [183]. This approach both minimizes contamination and provides a degree of cost-effectiveness and standardization that should make it ideal for rapid screening of high-risk hemato-oncology patients [95,164]. Indeed, sensitivity approaching 100% has been documented in prospective clinical studies, with two consecutive positive results being strongly associated with the development of IFI. Sequential PCR positivity has preceded both clinical signs and symptoms and clinical diagnosis by several days [95,156,157,159,164,165].

## 12. Clinical and Economic Impact of Rapid Diagnostic Tests in Candidasis and Cryptococcosis

Despite the fact that they are post-culture and thus delayed by 24–48 h, molecular and proteomic approaches for identification of *Candida* from positive cultures improve the time to identification when compared to conventional methods and may be useful in antifungal stewardship interventions promoting optimal antifungal therapy with the most cost-effective antifungal agents, resulting in improved outcomes and significant antifungal savings for hospitals. A cost minimization model was developed by Alexander *et al.* [78] to assess cost savings associated with implementation of the PNA-FISH test in a hospital with a rate of 40% for *C. albicans* candidemias and in which caspofungin was used over fluconazole for empiric treatment of IC. In their study, they predicted that the use of the PNA-FISH test would result in a cost savings of approximately $1,800 per patient from reduced caspofungin use (switch to fluconazole in patients infected with *C. albicans*), despite the fact that laboratory costs for doing the *C. albicans* PNA-FISH test ($82.72 per test) exceeded those for the *C. albicans* screen test ($2.83) or the Germ tube test ($4.42). A subsequent study at the University of Maryland [79] used clinical data to show the effect of PNA-FISH testing for *C. albicans* and validated the decision model of Alexander *et al.* [78]. The Maryland investigators found that 43% of candidemias in their hospital were due to *C. albicans* and that the most pronounced effects of the PNA-FISH test was on caspofungin usage in patients with *C. albicans* fungemia. In this group, there was significant reduction in the usage of caspofungin (shift to fluconazole) with a corresponding decrease in antifungal costs of $1,978 per patient [79]. The overall cost savings in reducing caspofungin usage surpassed the cost of the PNA-FISH test (net savings of $1,729 per patient) and led to the development of straightforward hospital-specific algorithms [79]. Additional studies have shown that post-PNA-FISH implementation, time to targeted therapy was significantly reduced [185,186,187], there was a significant improvement in culture clearance [185], and even when the cost of testing was factored in, there was an overall cost savings when treatment decisions were based on PNA-FISH results [185,186].

The ability to detect the presence of fungal pathogens directly in clinical specimens offers exciting new possibilities for the early diagnosis and management of IFIs in high risk patients [39,63,148,150]. A potential strategy for the use of non-culture-based methods for the diagnosis of IFI is to stratify patients according to risk and conduct prospective screening using the rapid test coupled with other diagnostic tests (e.g., cultures and imaging studies) [128,188,189,190,191]. This strategy should facilitate earlier species-level diagnosis thereby allowing aggressive pre-emptive antifungal therapy. A highly sensitive and specific test should also help to reduce empirical therapy with concomitant reduction in selection pressure for resistance as well as cost savings for the hospital [107,150,189,192,193].With these potential benefits in mind, two recent studies have assessed the potential of the T2Candida Panel to improve the diagnosis of candidemia and thereby quantify the economic and mortality impact of this new technology [194,195].

Aitken *et al.* [194] performed a prospective cohort study of patients with candidemia or receiving antifungal therapy in a university-affiliated tertiary care hospital. They examined the species of *Candida* identified, time to initiation of therapy, time to identification to species, and indications for antifungal use. Among a total of 162 patients with candidemia, the average time to yeast detection was 2.2 ± 1.3days with considerable variation according to species (range 0.6–7.9 days). The average time for initiation of antifungal therapy was 3.5 ± 2.1 days using an automated BC system and routine methods for species identification (*i.e.*, Vitek 2 and API 20c AUX [bioMerieux, Marcy l’Etoile France]). In 5000-patient Monte Carlo simulations the average time to initiation of antifungal therapy was 0.6 ± 0.2 days when the T2Candida Panel was used for detection and species-level identification directly from whole blood versus 2.6 ± 1.3 days and 2.5 ± 1.4 days, respectively, when either PNA-FISH (peptide nucleic acid fluorescent in situ hybridization) or MALDI-TOF MS (matrix-assisted laser desorption/ionization time of flight mass spectrometry) methods were used to identify *Candida* species from positive BCs.

The T2Candida Panel was further assessed as a tool for stopping unnecessary empiric echinocandin therapy. Among patients who received echinocandin therapy, 21% had a single negative BC and no subsequent evidence of *Candida* infection. Using this proportion as a base, two simulations were run where all 21% or only 11% of patients had echinocandin therapy discontinued within 0.3 ± 0.5 days based on a negative T2Candida result. Using a 5000-person Monte Carlo simulation, the average length of therapy was 5.8 ± 4.0 days using the T2Candida Panel with an 11% discontinuation rate, and 4.6 ± 4.0 days using the T2Candida Panel with a 21% discontinuation rate. In this 5000-person model, between 3136 and 6078 fewer doses of an echinocandin would be given based on an 11% or 21% discontinuation rate, respectively, with a potential cost savings of approximately $700,000 to $1.4 million per year using Red Book prices. Whereas the savings at a hospital level may vary depending on hospital contracting for echinocandins, volume of candidemia, cost of the T2Candida Panel, and the effectiveness of stewardship interventions, savings are still likely to be substantial. It should be noted that these projected savings depend on the active notification and intervention in a timely manner. In accordance with other authors [107,108], it is recognized that the clinical or economic impact of rapid diagnostic tests may be enhanced by an active stewardship effort. Thus, rapid detection and identification of *Candida* species directly from whole blood using the T2Candida Panel has the potential to direct early targeted therapy as well as to de-escalate unnecessary empirical antifungal therapy with resultant improved patient outcomes and reduced antifungal expense and selection pressure for resistance.

In a second study, Bilir *et al.* [195] developed a one-year decision—tree model to estimate hospital costs and effects (candidemia-related deaths) of using the T2Candida Panel versus BC alone, accounting for disease prevalence, distribution of *Candida* species, test characteristics (sensitivity, specificity, time to result), antifungal medication use, and differential length of stay (LOS) and mortality, by time to appropriate treatment initiation. The model assumes perfect (100%) sensitivity and specificity for BC with 3.6 to 6.4 days for detection and species identification, while assuming lower sensitivity (91.1%) and specificity (99.4%) for T2Candida with same day (3–5 h) results based on the results of the pivotal clinical trial [150]. Hospital costs for the management of candidemia were derived from an analysis performed using the IMS Hospital Charge Data Master (CDM) database. This database contains detailed hospital stay data collected annually for approximately 9 million inpatient encounters from over 650 hospitals. A retrospective cohort analysis of inpatient hospital billing records from 1 January 2011 through 30 September 2013 was conducted for adult (18 years and over) patients with a primary or secondary candidemia admission or discharge diagnosis (ICD-9: 112.5, 112.89, 112.9), at least one diagnostic test such as a BC, and evidence of antifungal therapy, to estimate candidemia-associated costs. A total of 3891 cases met inclusion criteria, of which 88.3% were diagnosed by BC. The CDM data, reported as charges, were converted using a cost-to-charge ratio derived from the publically-available Healthcare Utilization Project data set (www.hcupus.ahrq.gov/home.jsp). The base case results were calculated using the assumption that in a hospital admitting 5100 high risk patients per year [194], approximately 153 patients will be diagnosed with candidemia, assuming a 3% prevalence rate [196]. Based on current guidelines [61] and antifungal survey results [197] micafungin was selected as the default targeted therapy for all *Candida* species, and administered empirically to 40% of patients in the BC arm prior to results in the default analysis [198]. Pharmacy costs associated with unnecessary empiric therapy were estimated for non-candidemia patients.

Using these assumptions the annual cost for testing and treating the high-risk patients with the baseline BC-based strategy was estimated at $12,298,598 compared to $6,440,150 in the projected T2Candida-based diagnostic strategy for a potential annual savings of $5,858,448 (47.6%). The cost per tested patient decreased from $2,411 to $1,263 when adopting an institution-wide T2Candida testing strategy for a potential savings of $1,148 per tested patient. Savings were predominately due to the shortened hospital stay for survivors and lower hospital costs associated with the lower mortality rates in the T2Candida strategy [40,41].

The estimated potential savings per patient with candidemia was $26,887 a 48.8% reduction in hospital costs. Rapid species detection and identification and earlier treatment associated with the T2Candida strategy resulted in fewer deaths, reducing the overall mortality by 31.7 deaths (60.6%; BC, 52.3 deaths; T2Candida, 20.6 deaths). Given these improvements, a diagnostic strategy including T2Candida may be favored over BC alone in both cost-effectiveness/cost-avoidance and outcomes analysis.

This study demonstrates that T2Candida, a diagnostic panel to detect and identify *Candida* species in 3 to 5 h directly from whole blood, has the potential to significantly reduce costs and mortality rates in patients at high risk for candidemia. Using an economic model to quantify the potential economic and clinical outcomes associated with utilizing the T2Candida Panel as a primary diagnostic tool in a hospital’s high-risk population, shows that diagnosing patients sooner, prompting earlier targeted therapy with resultant decreases in mortality and LOS, may dramatically decrease hospital costs. The application of species-specific therapy enabled by rapid *Candida* identification demonstrated a potential savings of over 30 lives per year in a typical hospital setting, corresponding to a 60.6% reduction in mortality. Coupling this mortality reduction with cost savings shows that a T2Candida diagnostic strategy may markedly improve the management of candidemia compared to the standard of care BC strategy.

It is also clear that early treatment of AIDS patients with cryptococcal antigenemia is both cost effective and life saving [33,132,199,200,201]. Prospective screening of AIDS patients in Uganda, Rwanda, Mozambique and South Africa with the LFD for detection of cryptococcal antigenemia and pre-emptive treatment of antigen-positive individuals with fluconazole has been shown to be highly effective in preventing progression to CM [33,199,200]. This screen and treat approach was more cost-effective than routine standard of care at prevalence levels of ≥0.6% due to the avoidance of hospitalization for treatment of more severe disease [199,200]. Although this strategy has been employed with great success in Africa, it has not been adopted in high-resource areas such as the U.S. despite a prevalence of antigenemia of 2.9% among U.S. AIDS patients [33,132]. Such a strategy should be considered for integration in routine patient care practices in both low- and high-resource settings.

## 13. Summary and Conclusions

The opportunistic yeast pathogens now constitute one of the most important threats to the survival of immunocompromised hosts. There is little doubt that in addition to *C. albicans* and *C. neoformans*, a vast array of yeast-fungi, previously considered to be non-pathogenic, may serve as significant human pathogens. Recognition of these emerging fungal pathogens has resulted in a better understanding of their clinical presentation and response to the available therapeutic measures. Conventional laboratory-based methods for diagnosis of fungal infection remain useful but are often slow and lack sensitivity. The newer rapid, sensitive and specific culture-independent methods for the diagnosis of IC and CM offer great promise for improved diagnosis and management of these serious infections.

The continuing threat to public health posed by mycoses [1,9,202], combined with the severity of IFIs and the difficulty encountered in their treatment [72], requires a skilled laboratory workforce with the capacity to rapidly identify causative agents in time to institute effective antifungal therapy. Key findings of a cross-sectional survey of U.S. laboratories revealed a lack of ongoing training in mycology and a need for more clinically appropriate and cost-effective laboratory practices [202]. The increasing complexity of medical mycology practice underscores the need for laboratory staff to have access to high-quality and clinically relevant continuing education [202]. Currently, training is needed in basic isolation procedures and in advanced topics such as identification of problematic molds and yeasts, antifungal susceptibility testing, and in molecular methods for detection and identification of fungal pathogens [202,203].

Newer broad-spectrum antifungal agents may prove useful in the management of IFI but in turn may require more sensitive methods for diagnosing the infections, as well as for estimating the extent of disease, in order to significantly impact disease outcome. Broad application of both new and established antifungal agents may also select more resistant organisms from the vast pool of environmental opportunistic fungi. Such “emerging” fungal pathogens will pose yet another set of diagnostic and therapeutic challenges and will require that they are both visualized in tissue and identified in culture to truly define their pathogenesis and response to treatment.

Although progress in the diagnosis of mycotic infections has been made in the past 2 decades-including the development of BDG- and cryptococcal antigen-based assays, the PNA-FISH and MALDI-TOF MS approaches for rapid identification of *Candida* and *Cryptococcus* species from cultures, the introduction of rapid, sensitive and specific approaches, such as T2MR, for direct detection of *Candida* from whole blood—a great deal of work remains to be done. Future efforts must be directed toward standardizing and validating additional molecular assays for the diagnosis of fungal disease and expanding the availability of the new FDA-approved and CLSI-recommended tests [34]. Finally, the lack of training in medical mycology is a growing crisis in clinical microbiology laboratories [202,203]. It is impossible for laboratory personnel to provide the testing necessary to keep up with the changes in medical mycology without adequate training [204].
